# P-1666. Ensitrelvir (ESV) Population Pharmacokinetics (PK) in Nonhospitalized Adults with COVID-19

**DOI:** 10.1093/ofid/ofaf695.1840

**Published:** 2026-01-11

**Authors:** Sean Avedissian, Ukamaka O Modebelu, Nathaniel J Rhodes, Kara W Chew, Eric S Daar, David A Wohl, Joseph Eron, Simon Portsmouth, Takeki Uehara, Ryosuke Shimizu, David Smith, Judith S Currier, Annie Luetkemeyer, Courtney Fletcher

**Affiliations:** University of Nebraska Medical Center, Omaha, NE; UNMC, Omaha, NE; Midwestern University, Downers Grove, IL; David Geffen School of Medicine at University of California, Los Angeles, California; Lundquist Institute at Harbor-UCLA Medical Center, Torrance, CA; University of North Carolina at Chapel Hill School of Medicine, Chapel Hill, North Carolina; University of North Carolina at Chapel Hill School of Medicine, Chapel Hill, North Carolina; Shionogi Inc, Florham Park, NJ; SHIONOGI & CO., LTD., Osaka-shi, Osaka, Japan; Clinical Pharmacology & Pharmacokinetics, Project Management Department, Shionogi & Co., Ltd. Osaka, Japan, Osaka, Osaka, Japan; University of California, San Diego, San Diego, California; David Geffen School of Medicine at University of California, Los Angeles, California; University of California San Francisco, San Francisco, CA; University of Nebraska, Omaha, Nebraska

## Abstract

**Background:**

ESV is an investigational oral protease inhibitor for severe acute respiratory syndrome coronavirus-2 infection. SCORPIO-HR was a Phase 3 global trial in nonhospitalized adults with COVID-19 with or without risk factors for progression to severe disease. A population PK study was conducted in a subset of participants.

**Figure:**
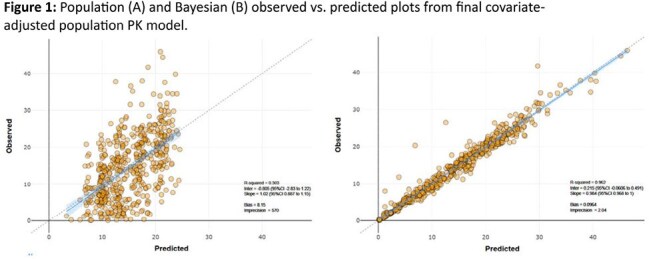

**Methods:**

Participants were randomized (1:1) to receive once-daily ESV (375 mg: Day 1, 125 mg: Days 2–5) or matching placebo. PK samples were collected on Days 1, 4, and 8 from n=400 participants. Population PK analysis used nonparametric methods (Pmetrics); covariates (age, sex, race, weight) were investigated.

**Results:**

PK samples were quantified from 222 participants randomized to ESV. Among them, 35 (16%) were excluded as all samples were < limit of quantitation (BLQ). An additional 21 (9.5%) were excluded for sporadic non-adherence (≥ 2 samples BLQ; n=8), as not evaluable, or inconsistent/not reported dose-sample collection times. The final data set was 166 participants with 579 detectable ESV levels. These 166 participants [n or mean (SD)] were: male, 106 (64%); age, 39.5 ± 13.8 yrs; weight, 68.4 ± 12.8 kg; Asian, 125 (75%); Black, 14 (8%); White, 25 (15%); unknown, 2. The final base model was an oral absorption (with lag), 2-compartmental model. Covariate analysis identified no race effect. In the final covariate model (Figure 1), there was an ≈13% effect on apparent clearance (CL/F) by sex, where CL/F in males was > females. Additionally, an effect on median central volume (Vc)/F by weight (centered to a 70kg person, 6.8L vs 7.3L) was found where Vc decreased as weight decreased and concentrations could increase. Covariate-adjusted final model PK estimates (median) were: CL/F, 0.313L/h; Vd/F, 18.66L; and terminal half-life 41h. Day 4 predose and 1.5h postdose mean (SD) concentrations were 17.5 (±5.8) and 20.5 (±6.7) µg/mL, respectively.

**Conclusion:**

ESV population PK parameters with covariate adjustment compared well with literature values from participants without COVID-19, indicating ESV PK disposition was not affected by COVID-19 in nonhospitalized symptomatic adults. The 16% of participants with all PK samples BLQ indicated no doses were taken and highlights the importance of objective adherence assessment in future COVID-19 studies.

**Disclosures:**

Nathaniel J. Rhodes, PharmD MS, Apothecademy, LLC: Advisor/Consultant Kara W. Chew, M.D., M.S., Pfizer: Medical writing support (no direct compensation) David A. Wohl, M.D., EMD Serono: Advisor/Consultant|EMD Serono: Honoraria|Gilead Sciences: Advisor/Consultant|Gilead Sciences: Honoraria|Merck: Advisor/Consultant|Merck: Honoraria|Regeneron: Advisor/Consultant|Regeneron: Honoraria|Theratechnologies: Advisor/Consultant|Theratechnologies: Honoraria|ViiV: Advisor/Consultant|ViiV: Honoraria Joseph Eron, MD, Gilead Sciences: Advisor/Consultant|Invivyd: DSMB|Merck: Advisor/Consultant Simon Portsmouth, MD, Shionogi Inc.: Employee Takeki Uehara, Ph.D., Shionogi, Inc.: Employee David Smith, MD, MAS, Bayer: Advisor/Consultant|Biosciences: Advisor/Consultant|Fluxergy: Stocks/Bonds (Private Company)|Gilead: Advisor/Consultant|Hyundai: Advisor/Consultant|IAS USA: Honoraria|Linear Therapies: Stocks/Bonds (Private Company)|Model Medicines: Advisor/Consultant|Model Medicines: Stocks/Bonds (Private Company)|NIH: Grant/Research Support|Pharma Holdings: Advisor/Consultant|Red Queen Therapeutics: Advisor/Consultant Judith S. Currier, MD, MSc, Merck and Co.: Honoraria Annie Luetkemeyer, MD, Cepheid: Grant/Research Support|Gilead: Grant/Research Support|GSK: Grant/Research Support|Merck: Grant/Research Support|ViiV: Grant/Research Support

